# Hit-hard and early versus step-up treatment in severe sarcoidosis

**DOI:** 10.1097/MCP.0000000000000906

**Published:** 2022-07-23

**Authors:** Adriane D.M. Vorselaars, Daniel A. Culver

**Affiliations:** a Division Heart and Lungs, University Medical Center Utrecht, Utrecht; b Interstitial Lung Diseases Center of Excellence, Department of Pulmonology, St Antonius Hospital, Nieuwegein, The Netherlands; c Department of Pulmonary Medicine, Respiratory Institute; d Department of Inflammation and Immunity, Lerner Research Institute, Cleveland Clinic, Cleveland, Ohio, USA

**Keywords:** hit-hard, sarcoidosis, step-up, top-down, treatment

## Abstract

The treatment of sarcoidosis remains uncertain, despite 70 years of study. The conventional approach is to initiate corticosteroids in individuals who require treatment. The position of more aggressive regimes is unknown.

**Recent findings:**

Recent recognition that many patients will require prolonged therapy, and the observation that corticosteroids lead to overt and insidious toxicities, have led to suggestions that steroid-sparing medications be used earlier in the management of sarcoidosis. Individuals with poor prognostic features, designated as ‘high-risk’ sarcoidosis may, especially benefit from a broader palette of therapeutic options in the initial treatment regimen. An even more aggressive approach, known as ‘top-down’ or ‘hit-hard and early’ therapy has emerged in the fields of gastroenterology and rheumatology in the past 15 years, on the premise that highly effective early control of inflammation leads to better outcomes. These regimens typically involve early initiation of biologic therapies.

**Summary:**

For certain subpopulations of sarcoidosis patients, ‘top-down’ therapy could be helpful. Severe pulmonary sarcoidosis, neurosarcoidosis, cardiac sarcoidosis and multiorgan sarcoidosis are phenotypes that may be most relevant for revised therapeutic algorithms. Precision medicine approaches and randomized trials will be necessary to confirm a role for top-down therapy in the routine management of sarcoidosis.

## INTRODUCTION: CURRENT THERAPEUTIC APPROACH IN SARCOIDOSIS

Corticosteroids have been used for sarcoidosis for over 70 years. Even now, they remain the most commonly used medication for systemic management [1^▪^]. Corticosteroids are highly effective, inexpensive, easily titrated, work quickly, are perceived to require less monitoring than other agents and are familiar to patients as well as prescribers. They have the added advantage in some patients of increasing energy, sense of wellbeing and ameliorating nonspecific symptoms such as arthralgias, at least when first prescribed. In addition, corticosteroids have been studied in various sarcoidoses over the past 6 decades [2]. As such, corticosteroids are still deemed by expert opinion to be the first-line therapy for all forms of sarcoidosis that require systemic treatment [3^▪▪^]. It should be noted that these opinions are based not on controlled data but rather on the absence of head-to-head comparisons between corticosteroids and other agents.

Although sarcoidosis experts typically advocate for earlier inclusion of steroid-sparing agents in treatment algorithms [4^▪^], in practice steroid-sparing agents are prescribed for much less than half of treatment-requiring patients [5,6]. Long-term management typically entails higher prednisone doses than the commonly referenced prednisone goal of less than 10 mg/day; for example the mean daily dose in two randomized pulmonary sarcoidosis trials was approximately 13 mg/day [7]. In an unselected cohort from our institution, the median treatment duration among all treated patients was 8 months [7]. These data accord with a multicenter prospective observational study in the United States that demonstrated that the likelihood of requiring treatment after 2 years in patients requiring treatment is approximately 50% [8,9]. In corticosteroid-treated patients, relapses after tapering of corticosteroids have been noted in 20–74% [10–12]. For those individual requiring tumor necrosis factor inhibitors, the likelihood of very early relapse after stopping therapy is especially high [13]. Taken as a whole, the available data paint a picture of frequent treatment initiation with corticosteroids but a failure of clinicians to reduce the corticosteroid dose by sufficiently aggressive use of steroid-sparing medications. This practice gap is likely due to uneven implementation of recommended steroid-sparing strategies, patient preference, access to medication, lack of knowledge, or the absence of sufficiently effective steroid-sparing agents. 

**Box 1:**
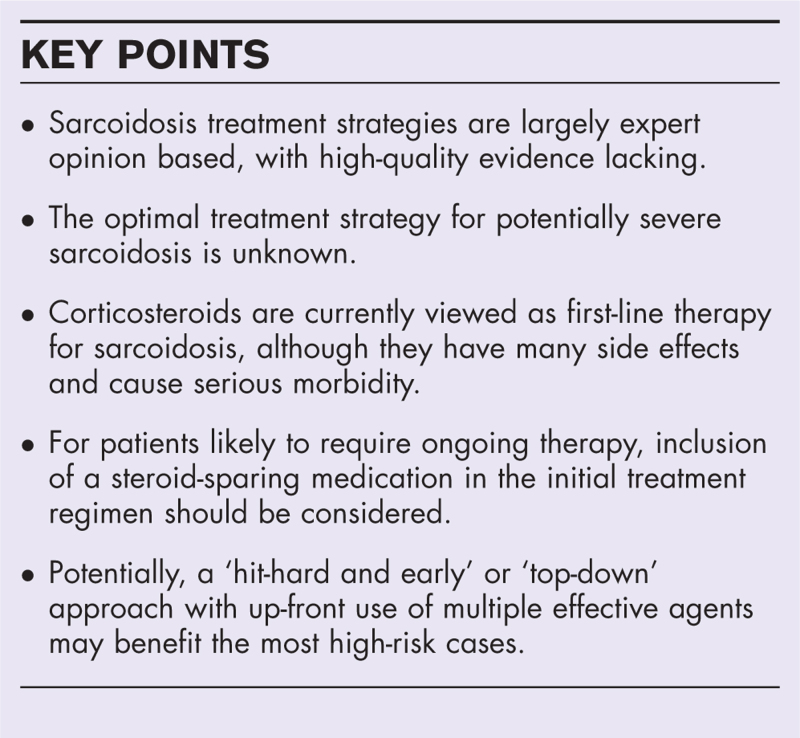
no caption available

## RATIONALE FOR HIT HARD AND EARLY TREATMENT

### Not always a benign disease

Sarcoidosis is an extremely heterogeneous disease – whereas many patients enjoy spontaneous resolution, a quantitatively important subset experience severe morbidity, loss of quality of life, or even death as a result. Most of these patients with poor outcomes exhibit chronic, nonself-limiting sarcoidosis, ongoing granulomatous inflammation and sometimes fibrosis. Uncontrolled inflammation is believed to be the instigator of fibrotic sarcoidosis, perhaps in the context of profibrotic genetic features and immune responses [14]. For pulmonary disease, the latency from presentation to the development of disability was over 100 months in one Japanese cohort, emphasizing the slow build-up of fibrosis and the need to view management decisions through a chronic treatment lens [15].

Compared with the general population, sarcoidosis leads to increased mortality [16–19]. A large population based study in Sweden, including over 8000 sarcoidosis patients revealed that those with sarcoidosis are at a higher risk of death compared with the overall population [16]. Moreover, patients with an indication for systemic treatment had a two-fold increased risk of death. A recent Danish study suggested that mortality rates in sarcoidosis patients are higher than controls for all age groups and sexes and are highest in patients treated with corticosteroids [20^▪^]. For pulmonary disease, death occurs mainly in those with fibrosis [18]. On the contrary, mortality rates in the United States and in Europe appear to be increasing [17,19]. In addition to pulmonary disease, cardiac involvement, neurologic involvement and multiorgan sarcoidosis are most closely associated with poor outcomes [15,21]. Disability and death were closely tied to multiple organ involvement at disease outset in a Japanese cohort, with three or more overtly involved organs at presentation delineating a substantial increased risk for poor outcome [15]. Other features can also be used to identify patients at higher risk, including older age at diagnosis, black race, lower socioeconomic status, extrapulmonary involvement, advanced cardio/pulmonary disease and pulmonary hypertension [22–25].

### Role of immunomodulators and biologics in sarcoidosis

**FIGURE 1:**
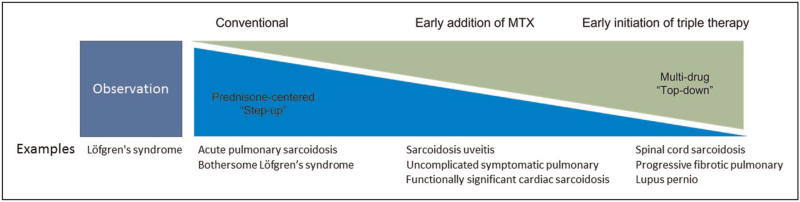
Proposed view on treatment in sarcoidosis. Sarcoidosis treatment strategies vary from observation or watchful waiting in cases without organ damage or severe diminished quality of life, for example uncomplicated Lofgren's syndrome to strategies with more upfront use of steroid sparing agents or even biologics for example cardiac or neurosarcoidosis.

First, a decision about whether to treat sarcoidosis must be established [26^▪^]. In patients without organ damage or severely impaired quality of life the decision not to treat can be made [26^▪^,^27^^▪▪^]. Patients in definite need for treatment are those with severe organ dysfunction such as severe pulmonary sarcoidosis, significant hypercalcemia, clinically important cardiac sarcoidosis or clinically important neurosarcoidosis [2,22], Fig. 1. It is estimated that approximately half of all sarcoidosis patients need systemic treatment [9].

As mentioned previously, the initial need for treatment is a predictor for mortality and prolonged treatment duration [16]. However, the use of corticosteroids portends (often) unsuspected long-term consequences that can ultimately worsen outcomes. For example, initiation of corticosteroids has been associated with side effects such as diabetes, obesity, osteoporosis and infections [7]. A large Swedish register study found a hazard ratio of 2.4 of developing type 2 diabetes in corticosteroid-treated sarcoidosis patients versus controls [28]. Furthermore, the dose of prednisone has been directly correlated with the amount of weight gain in sarcoidosis patients, without an extra beneficial effect on lung function with higher doses [29]. The initiation of therapy has been associated with a higher risk for myocardial infarction, a potential consequence of steroid-related toxicities [30^▪^]. Even in the short term, patients receiving higher doses of steroids had a lower health related quality of life compared with patients on lower doses, when adjusting for disease severity [31]. Thus, there is a growing recognition of significant morbidity and mortality due to steroid use in sarcoidosis, and an appetite for earlier implementation of steroid sparing regimens [32]. This sentiment is reflected in the recent ERS Treatment Guidelines, which suggest consideration of a steroid-sparing medication at disease outset in cardiac sarcoidosis, but which stopped short of suggesting it for all treatment-requiring patients with cardiac sarcoidosis due to the lack of robust evidence [3^▪▪^].

Immunomodulatory drugs such as methotrexate, azathioprine and leflunomide can be used as steroid sparing options in sarcoidosis, with methotrexate being used most often [4^▪^,^33^,^34^]. Methotrexate was recently found to be effective in up to 80% of pulmonary sarcoidosis patients [35]. However, an upfront approach for methotrexate or other steroid sparing medications in sarcoidosis is not yet supported by rigorous evidence. A Delphi consensus study on sarcoidosis treatment revealed large variations in treatment regimens but emphasized the use of immunomodulators such as methotrexate in disease likely to require prolonged treatment, or as a steroid-sparing option in patients with high risk of steroid toxicity [4^▪^]. The Delphi study does not provide firm guidance on exactly when to initiate methotrexate (e.g., upfront as monotherapy, upfront together with steroids or after inability to taper steroids). However, it is notable that a patient reported outcome study in sarcoidosis patients reported side effects in 78% of prednisone treated patients versus 49% of methotrexate-treated patients [36].

Significantly, a recent cohort study compared pulmonary sarcoidosis patients treated with first-line methylprednisone or methotrexate [37]. Patients receiving methotrexate as first-line monotherapy had contraindications for corticosteroids. The data suggest that effectiveness in pulmonary sarcoidosis was noninferior in the methotrexate group, with indications of lower rates of treatment resistance and relapse. Similarly, a small cohort study in cardiac sarcoidosis showed that methotrexate and corticosteroid combination therapy had better results on left ventricular function than corticosteroids alone [38]. To date, there are no published data from randomized trials comparing up-front corticosteroid versus steroid-sparing monotherapy approaches.

Biologics such as tumor necrosis factor inhibitors (TNFi) drugs have mostly been reserved for severe refractory sarcoidosis cases, as outlined in the ERS Guideline [3^▪▪^]. The ERS guideline provides a conditional recommendation with low-quality evidence for their use [3^▪▪^]. High disease activity on 18-fluorodeoxyglucose (FDG)-PET correlates with effectiveness of TNFi [39], but routine PET has not been sufficiently established to use as a gating criterion for the decision to start TNFi. Although TNFi are considered off-label for sarcoidosis, a Delphi consensus study amongst global sarcoidosis experts revealed consensus for their use in common practice for severe cases [4^▪^]. Whether to initiate TNFi only after progressing through other agents first is controversial, as they are remarkably effective for certain indications that otherwise are extremely difficult to control, such as neurosarcoidosis and lupus pernio [40,41]. However, there are no randomized trials in sarcoidosis comparing immediate use of TNFi versus the conventional step-up approach. Potentially, earlier use could better preserve organ function and diminish morbidity. A large cohort study comparing treatment outcomes of infliximab and methotrexate in sarcoidosis patients revealed that infliximab was more likely to improve clinical status after 1 year than methotrexate [42^▪^]. A high variability in biologic use for sarcoidosis currently exists globally, but even within countries, potentially due to local preferences, physician experience and also reimbursement. Data From the American College of Rheumatology's Rheumatology Informatics System for Effectiveness Registry showed that even in practices with over 30 sarcoidosis patients, biologic use ranged from 16 to 67% [43^▪^].

In the current guidelines, biologics are reserved for cases proven refractory to first-line and second-line therapy which often leads to a potential suspension of months to years until patients receive biologic treatment. In case of high-risk sarcoidosis it is unknown whether this is harmful. Two cohort studies of infliximab and inflectra treated patients showed a decrease in lung function while on first-line or second-line treatment in the 6 months prior to biologic therapy [39,44]. For spinal sarcoidosis, delay of effective treatment has been associated with poorer outcomes [45], and the use of TNFi resulted in earlier disease control with better outcomes [46]. Similarly, delay in effective therapy for cardiac sarcoidosis has been associated with lower chance for restoration of intrinsic conduction and cardiac function [47,48]. Obviously, all of these observational studies are fraught with methodologic pitfalls, rendering inferences from them extremely tenuous. Nonetheless, they are all concordant with the hypothesis that earlier use of highly effective therapy may result in better outcomes. Examples of low-risk versus high-risk sarcoidosis are mentioned in Table 1.

**Table 1:** Examples of sarcoidosis manifestations and risk stratification

Low-risk sarcoidosis	Intermediate-risk sarcoidosis	High-risk sarcoidosis • Factors warranting intensive treatment
Lofgrens syndrome		
Nodal sarcoidosis		
Scar sarcoidosis		
(Ankle) arthritis	Osseous sarcoidosis	
Uncomplicated or local cutaneous involvement		Lupus Pernio *(known to warrant long-term treatment)*
	Uncomplicated uveitis	Chronic or severe uveitis • Loss of vision
	Mild hypercalcemia hypercalciuria with nefrocalcinosis	Severe hypercalcemia *(known to warrant long-term treatment)*
	Possible cardiac sarcoidosis	Probable or definite cardiac sarcoidosis • Large extent of involvement on CMR or FDG-PET • Rhythm or function abnormalities
	Parenchymal sarcoidosis without impaired PFT	Progressive fibrosing pulmonary sarcoidosis • Impaired pulmonary function • High inflammatory burden and signs of fibrosis on FDG-PET/CT
		Central nervous system sarcoidosis • Spinal cord involvement • Hydrocephalus
	Hepatic sarcoidosis	

Simplified stratification of sarcoidosis examples into risk categories. Risk of organ damage or mortality are taken into account. Low-risk sarcoidosis can be managed by watchful waiting and screening for organ involvement. Intermediate-risk sarcoidosis should be managed case-by-case, some patients warrant systemic treatment following current guidelines with a step-up regime. High-risk sarcoidosis warrants systemic treatment and could potentially benefit from a more aggressive ‘top-down’ or ‘hit-hard’ regimen with a more upfront use of steroid sparing agents or in some cases even biologicals. CMR Cardiac Magnetic Resonance Imaging; FDG, fluorodeoxyglucose; PFT, pulmonary function test.

### Hit-hard and early, evidence in rheumatology and inflammatory bowel disease

In Crohn's disease, a granulomatous inflammatory bowel disease (IBD), the use of top-down versus step-up treatment has been a subject of investigation in the last decade with promising results for a combination therapy of immunomodulators and biologicals for severe disease [49]. A large meta-analysis favored biologic and azathioprine combination therapy as induction therapy above immunosuppressants alone [50]. Furthermore, in Crohn's disease earlier introduction (within 3 months of diagnosis) of TNFi tended to correlate with the slower progression of long-term bowel damage [51]. The British Society of Gastroenterology consensus guidelines recommend escalation to an alternative agent in refractory patients after 8 weeks of therapy, and suggest even earlier use of biologic agents in patients with poor prognostic features [52]. Importantly, the British Guideline also strongly recommends against monotherapy with corticosteroids for maintenance of remission, due in part to therapeutic toxicity [52].

Similar to IBD, current management philosophy in rheumatoid arthritis also emphasizes earlier disease control. The European League Against Rheumatism endorses the immediate initiation of methotrexate or another immunomodulator (with or without corticosteroids) to diminish the chance of permanent joint damage at the time of diagnosis [53]. After 3 months an improvement should be seen, otherwise a biologic should be introduced. When after 6 months the established treatment target is not reached, a biologic (such as TNFi) should be introduced [53]. This principle of hit-hard and early with methotrexate up-front and tight control with biologics after a maximum of 3–6 months from diagnosis when needed could potentially be extrapolated to severe ‘High risk’ sarcoidosis patients. Of course, sarcoidosis patients can also experience spontaneous remission, so this strategy requires careful periodic reassessment and will benefit from precision medicine approaches to prognosis and treatment.

## GAP OF EVIDENCE IN THE CURRENT LITERATURE

Sarcoidosis is already an orphan disease, with a treatment-requiring proportion of approximately 50%. The category of patients truly refractory to standard first/second-line treatment is even smaller, but ‘refractory’ is an arbitrary term that may overestimate how many patients can be managed with tolerable or minimally toxic doses of corticosteroids. It is estimated that 10–30% of patients develop progressive pulmonary disease [54]. Due to small numbers of patients with severe disease, most available evidence, outside corticosteroids, comes from small randomized controlled trials (RCTs), retrospective cohort studies and expert opinion. Although some pharmaceutical RCTs have been performed in sarcoidosis in the last 2 decades, these have mostly been investigating new drugs or sarcoidosis as a new target disease. Due to the absence of regulatory approval and difficulty with access, these medications are at this moment not replacing long-standing therapies. There is also an evidence gap in high-quality RCTs for effective conventional therapies due to the lack of interest in funding such trials for generic medications. At the moment of writing this article, clinicaltrials.gov mentions worldwide only nine treatment related trials in any form of sarcoidosis currently recruiting patients (including one in ocular, one in hepatic sarcoidosis and two treatment trials in sarcoidosis-associated pulmonary hypertension).

A highlight related to this particular article is that two extant trials are currently investigating the role of methotrexate as a substitute for initial treatment of sarcoidosis. The PREDMETH trial is a multicenter RCT currently enrolling patients with pulmonary sarcoidosis and impaired lung function in the Netherlands [55^▪^]. This study intents to investigate the efficacy and tolerability of methotrexate as first-line therapy with the hypothesis that methotrexate is just as effective as prednisone treatment, but with fewer side effects. Another currently enrolling treatment-related RCT is CHASM-CS-RCT [56^▪^]. This multicenter RCT is designed to evaluate the optimal first-line treatment strategy for patients with active cardiac sarcoidosis. The hypothesis is that a low-dose prednisone/methotrexate combination will not have inferior efficacy to first-line prednisone treatment but will have a reduced burden of side effects resulting in a better quality of life.

Besides RCTs, the use of (global) registries with well phenotyped patients is a critical first step to systematically study high-risk or severe sarcoidosis and the optimal treatment regiments. The marginal benefit of registries compared with RCTs is the option to include a more heterogeneous (and more representative) sarcoidosis population, gain large numbers and also gain insight in the use of off-label biologics.

## CONCLUSION

With the recent publication of the ERS and BTS guidelines on sarcoidosis management the gap of knowledge and paucity of rigorous evidence in this field becomes strikingly clear. However, these guidelines do provide consistency of direction for physicians treating sarcoidosis patients. A step-up approach with initial corticosteroid monotherapy is favored by the ERS/BTS guideline, although some other reviews or expert opinions leave room for initiation of upfront second-line treatment (e.g. methotrexate) together with steroids in severely endangered or ‘High risk’ sarcoidosis patients.

Patient selection will always pose a significant challenge, because not all patients need systemic treatment and all available treatment options have potential side effects. The optimal treatment strategy for these severe, sometimes even life threatening, sarcoidosis cases is unknown. These cases include severe pulmonary, cardiac and neurosarcoidosis. Potentially, extrapolating literature from other disciplines, a ‘hit-hard and early’ approach could benefit severe life or organ threatening sarcoidosis for which a prolonged treatment regimen is anticipated. Evidence from ongoing and future trials in sarcoidosis treatment are needed.

## Acknowledgements

*None.*

### Financial support and sponsorship

*The current work was not supported or sponsored.*

### Conflicts of interest

*A.D.M.V. received consulting fees from Boehringer Ingelheim. D.A.C. received grant support from Boehringer-Ingelheim, Mallinkrodt, AI therapeutics, aTyr Pharma and Janssen. Personal consulting fees, steering committee or travel fees from aTyr Pharma, Krevant, Roche, Boehringer-Ingelheim, Fibrogen and Pliant.*

## REFERENCES AND RECOMMENDED READING

Papers of particular interest, published within the annual period of review, have been highlighted as:

▪ of special interest
▪▪ of outstanding interest
